# The Small Toxic *Salmonella* Protein TimP Targets the Cytoplasmic Membrane and Is Repressed by the Small RNA TimR

**DOI:** 10.1128/mBio.01659-20

**Published:** 2020-11-10

**Authors:** Liis Andresen, Yolanda Martínez-Burgo, Josefin Nilsson Zangelin, Alisa Rizvanovic, Erik Holmqvist

**Affiliations:** a Department of Cell and Molecular Biology, Biomedical Centre, Uppsala University, Uppsala, Sweden; National Institute of Child Health and Human Development (NICHD)

**Keywords:** small protein, toxin-antitoxin, TA system, sRNA, growth inhibition, *ryfA*, membrane stress, posttranscriptional control, small RNA

## Abstract

Small proteins are gaining increased attention due to their important functions in major biological processes throughout the domains of life. However, their small size and low sequence conservation make them difficult to identify. It is therefore not surprising that enterobacterial *ryfA* has escaped identification as a small protein coding gene for nearly 2 decades. Since its identification in 2001, *ryfA* has been thought to encode a noncoding RNA and has been implicated in biofilm formation in Escherichia coli and pathogenesis in Shigella dysenteriae. Although a recent ribosome profiling study suggested *ryfA* to be translated, the corresponding protein product was not detected. In this study, we provide evidence that *ryfA* encodes a small toxic inner membrane protein, TimP, overexpression of which causes cytoplasmic membrane leakage. TimP carries an N-terminal signal sequence, indicating that its membrane localization is Sec-dependent. Expression of TimP is repressed by the small RNA (sRNA) TimR, which base pairs with the *timP* mRNA to inhibit its translation. In contrast to overexpression, endogenous expression of TimP upon *timR* deletion permits cell growth, possibly indicating a toxicity-independent function in the bacterial membrane.

## Abstract (precis)

Next-generation sequencing (NGS) has enabled the revelation of a vast number of genomes from organisms spanning all domains of life. To reduce complexity when new genome sequences are annotated, open reading frames (ORFs) shorter than 50 codons in length are generally omitted. However, it has recently become evident that this procedure sorts away ORFs encoding small proteins of high biological significance. For instance, tailored small protein identification approaches have shown that bacteria encode numerous small proteins with important physiological functions. As the number of predicted small ORFs increase, it becomes important to characterize the corresponding proteins. In this study, we discovered a conserved but previously overlooked small enterobacterial protein. We show that this protein, which we dubbed TimP, is a potent toxin that inhibits bacterial growth by targeting the cell membrane. Toxicity is relieved by a small regulatory RNA, which binds the toxin mRNA to inhibit toxin synthesis.

## INTRODUCTION

Annotation of small open reading frames (sORFs) in genomic sequences is challenging because they are indistinguishable from numerous small nonfunctional in-frame genome fragments. To reduce this unwanted background, most gene prediction tools apply ORF length cutoffs, which, however, creates a bias toward annotation of longer ORFs and exclusion of sORFs shorter than 50 codons in prokaryotic and 100 codons in eukaryotic genomes. In the last decade, this systematic bias has been acknowledged, and impressive progress has been made in the field of sORF identification by combining advanced computational prediction with experimental methods (1–13), recently reviewed for Escherichia coli in reference 14. These studies demonstrate that small protein genes are much more abundant than previously imagined. For instance, more than 100 sORFs have been experimentally verified in the model organism E. coli (3, 4, 12, 14, 15). An extensive metagenomics study of the human microbiome identified more than 4,000 putative small protein families, indicating a hidden world of small proteins awaiting to be explored (10). However, since characterization of small proteins has only recently begun, the functions of most putative small proteins are currently unknown.

Before the era of genome-wide discovery of small protein genes, case-by-case discovery over the years has shown that, as with their larger counterparts, small proteins have important functions throughout the domains of life. Small proteins play essential roles in organismal development and carry out niche- or tissue-specific functions (for examples, see references [Bibr B16][Bibr B17][Bibr B18]). In bacteria, small proteins participate in central cellular processes by being components of ribosomes, cytochrome oxidase complexes, or the cell division apparatus (19, 20). They can also act as regulators of specific transporters (21–25) or signal transduction pathways (26, 27). A special class of bacterial small proteins are toxins in type I toxin-antitoxin (TA) systems.

In E. coli, most type I toxins are between 18 and 51 amino acids in length, with the IbsB toxin being the smallest and HokD the largest within this size range. As a common feature, these small proteins are toxic upon overexpression, resulting in growth arrest (for a review on TA systems, see reference 28). The antitoxins of type I TA systems are antisense RNAs, which are transcribed from a sequence overlapping, or located adjacent to, the toxin gene (29). Antitoxin RNAs base pair to their respective toxin mRNAs to inhibit translation and/or to induce mRNA degradation (30). The antitoxins are generally more labile than toxin mRNAs. It has therefore been suggested that the toxin can affect cells under physiological conditions in which antitoxin synthesis is stopped and/or the antitoxin is degraded (28). Since type I toxin translation is generally repressed during growth in common laboratory media, most research on these systems has been done with ectopic expression of the system components. These studies have shown that, when overexpressed from a plasmid, type I toxins damage the cells in different ways, often by compromising the cytoplasmic membrane (31). This occurs either through toxin oligomerization and pore formation in the membrane, leading to membrane depolarization and leakage (32, 33), by interference with membrane synthesis, or by disruption of membrane organization (33). Membrane-damaging type I toxins are thought to insert directly into the membrane, without the help of a membrane insertion machinery such as the Sec system. However, although most type I toxins are small hydrophobic proteins targeting the membrane, SymE and RalR are exceptions to this rule, as they appear to act as nucleases to mediate toxicity (34, 35). While molecular mechanistic details of TA systems have been studied in detail, their biological functions are less understood. It has been reported that TA systems induce cell death under unfavorable conditions (e.g., postsegregational killing and abortive infection) or that controlled activation of toxins can induce a transient state of dormancy that promotes stress tolerance (28).

Here, we describe the discovery of a toxic protein-coding gene and its antisense repressor encoded in the genomic region of the *ryfA* gene in Salmonella enterica serovar Typhimurium. We show that although *ryfA* was initially annotated to encode a noncoding RNA (36), it contains a small ORF. This ORF is translated into a 38-amino-acid small protein that is toxic upon overexpression. The protein harbors a canonical signal sequence and is localized in the cytoplasmic membrane. Toxicity is repressed by a small RNA (sRNA) encoded divergently from *ryfA*, a gene arrangement resembling that of type I TA systems. Based on the results presented in this study, we suggest renaming *ryfA* to *timP* (toxic inner membrane protein) and its repressor sRNA gene to *timR* (*timP* repressor).

## RESULTS

### Overexpression of *timP* leads to growth inhibition.

**FIG 1:**
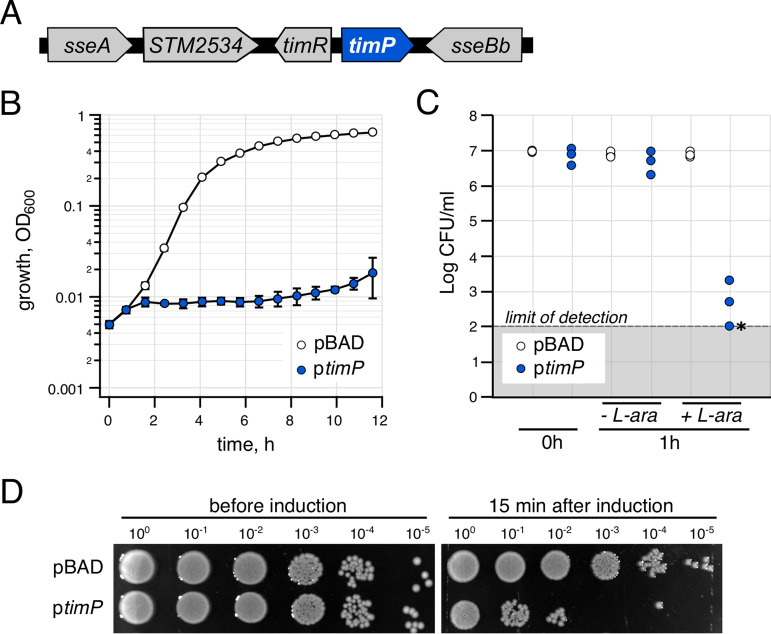
TimP overexpression inhibits growth of *Salmonella* serovar Typhimurium. (A) Genomic context of the *timP* gene in *Salmonella* serovar Typhimurium strain SL1344. (B) Growth curves of wild-type cells carrying an empty control vector (pBAD33) or an arabinose-inducible *timP* overexpression construct (pYMB023) in M9 minimal medium supplemented with 0.4% glycerol, 0.1% Casamino Acids, and 0.2% l-arabinose. Error bars indicate standard deviations (SD) from 3 independent transformants. Optical density was monitored at 600 nm during growth in 96-well plates. (C) CFU counts of bacteria exposed to l-arabinose (0.2% final concentration) for 1 h to induce *timP* expression, followed by washing, dilution, and plating on inducer-free agar plates. Each dot represents a result obtained from an individual transformant. The asterisk indicates a sample where no colonies were detected. (D) Spotting assay on inducer-free plates after exponentially growing cultures were exposed to 0.2% l-arabinose for 15 min.

The *timP* gene (formerly *ryfA*) in E. coli K-12, located between *sseA* (encoding 3-mercaptopyruvate sulfurtransferase) and *sseB* (serine sensitivity enhancing B), was initially proposed to encode a 300-nucleotide (nt)-long noncoding RNA (36). Homologs of this RNA have been associated with virulence in *Shigella* (37) and biofilm formation in pathogenic E. coli (38). The genomic context of *timP* in *Salmonella* serovar Typhimurium strain SL1344 (henceforth *Salmonella*) differs from that of K-12 in that a gene of unknown function, *STM2534*, is annotated between *sseA* and *timP.* In addition, the uncharacterized sRNA gene *timR* (formerly *STnc2070*) is annotated next to *timP* (Fig. 1A; see also Fig. S1 in the supplemental material). During our first experiments aimed at characterizing *timP* function in *Salmonella*, we observed that its overexpression from an inducible promoter strongly inhibited bacterial growth. During growth in 96-well plates in medium containing the inducer, the optical density (OD) of the *timP* overexpression strain showed almost no increase during a 12-h incubation period (Fig. 1B). Similarly, spotting dilutions of bacterial cultures on plates containing the inducer resulted in a strong reduction in the number of CFU when cells harbored the *timP*-inducible plasmid (Fig. S2). The observed growth inhibition may be due either to a temporary growth arrest that would allow cells to recover after removing the inducer or to a toxic effect that permanently damages the cells. To test this, overnight cultures were diluted 300-fold in medium containing the inducer for 1 h, after which *timP* expression was repressed by washing the bacteria in medium lacking the inducer and plating serial dilutions on inducer-free plates. A 1-h induction of *timP* resulted in a strong decrease (4 orders of magnitude) in the number of CFU, suggesting that *timP* overexpression causes irreversible cell damage that prevents growth resumption (Fig. 1C). Finally, to test whether *timP* overexpression can inhibit actively growing cells, cultures were grown to mid-exponential phase, after which the inducer was added for 15 min, followed by washes and plating on inducer-free plates. As shown in Fig. 1D, the 15-min pulse of *timP* expression reduced viability by 3 orders of magnitude, indicating that actively growing cells are highly sensitive to *timP* overexpression.

10.1128/mBio.01659-20.5 FIG S1 Nucleotide sequence of the *timR-timP* locus in *Salmonella* serovar Typhimurium. Download FIG S1, EPS file, 0.1 MB. Copyright © 2020 Andresen et al. 2020 Andresen et al. This content is distributed under the terms of the Creative Commons Attribution 4.0 International license.

10.1128/mBio.01659-20.6 FIG S2 Addition of a histidine tag in the TimP C terminus does not affect toxicity. A *Salmonella* Δ*timP* strain was transformed with plasmid constructs carrying the *timP* ORF C-terminally fused to the indicated affinity tags. Cells were grown overnight in LB medium, and serial dilutions of the culture were spotted on either LB plates (non-induced) or LB plates containing 0.2% l-arabinose (induced). Download FIG S2, EPS file, 0.5 MB. Copyright © 2020 Andresen et al. 2020 Andresen et al. This content is distributed under the terms of the Creative Commons Attribution 4.0 International license.

### The *timP* gene encodes a small protein.

**FIG 2:**
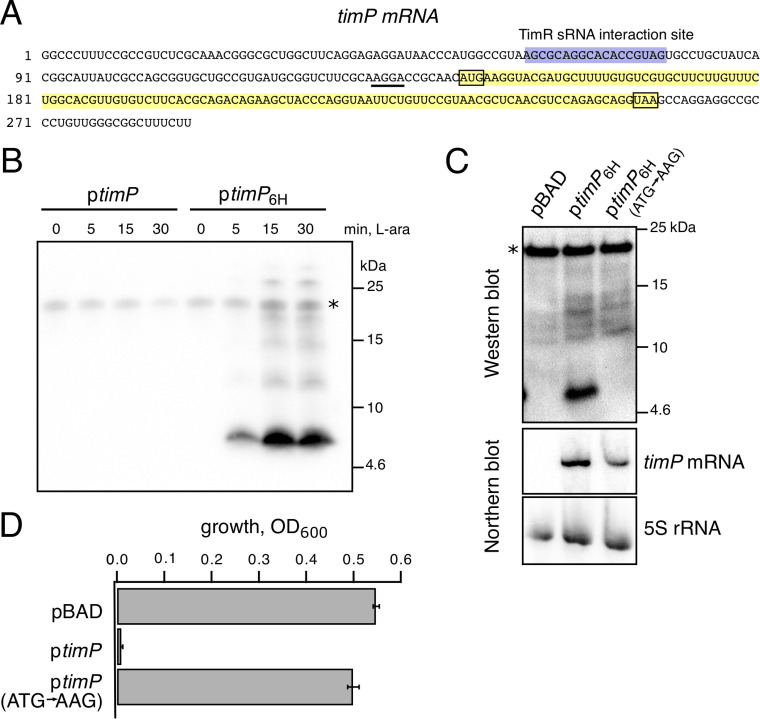
The *timP* gene encodes a small toxic protein. (A) Sequence of the *timP* mRNA. The predicted ORF is indicated in yellow, a putative Shine-Dalgarno sequence is underlined, and the TimR binding site is highlighted in purple. (B) The ORF shown in panel A was C-terminally tagged with six histidine residues on the arabinose-inducible *timP* overexpression construct (pYMB023 → pLA208). Wild-type *Salmonella* cells harboring the *timP-6×His* plasmid or the parental nontagged plasmid were grown in M9 medium. At an OD_600_ of 0.3, l-arabinose was added to the cultures to induce *timP* expression. Before induction and after 5, 15, and 30 min, cells were harvested for immobilized metal affinity chromatography and Western blotting. The asterisk indicates an unspecific signal which serves as loading control. (C) The start codon of the *timP* ORF was mutated (ATG to AAG) on the arabinose-inducible *timP-6×His* overexpression construct. Strains carrying either of the plasmids pBAD (vector control), pLA208 (*timP-6×His*), or pLA218 [*timP(ATG*→*AAG)-6×His*] were grown to exponential phase in M9-glycerol medium. After 15 min of induction with 0.2% l-arabinose, cells were harvested for *timP* expression detection by Western and Northern blotting. The asterisk indicates an unspecific signal which serves as loading control. (D) The growth of *Salmonella* carrying either the control vector, the *timP* overexpression plasmid (pYMB023), or the *timP* start codon mutant plasmid (pYMB024) was measured in M9-based medium supplemented with 0.2% l-arabinose for *timP* induction. Bars indicate the optical densities of the cultures 8 h after inoculation (averages from three independent transformants ± SD).

Although *timP* had been suggested to encode a noncoding RNA (36), recent ribosome profiling data indicated that it might encode a small protein (designated mia-62 in reference 39). In agreement with this, running the RNAcode software (40) on the *timP* sequence alignment available in Rfam (*ryfA* family, RF00126) predicted a conserved ORF spanning nt +145 to +261 relative to the *timP* transcription start site (Fig. 2A; Fig. S3) (39). In order to test whether the predicted ORF was translated *in vivo*, a hexahistidine tag-encoding sequence (*6×His*) was inserted directly before the ORF’s stop codon in the *timP* overexpression construct. Western blot analysis using an anti-His probe confirmed the expression of the 5-kDa TimP protein, which started to accumulate by 5 min after addition of the inducer (Fig. 2B). Importantly, addition of the histidine tag did not impair the toxicity of TimP overexpression, whereas several other tested tags strongly reduced toxicity (Fig. S2). A start codon mutation (ATG to AAG) in the *timP* ORF completely abolished TimP synthesis without significantly affecting *timP* mRNA levels (Fig. 2C) and rendered *timP* overexpression nontoxic (Fig. 2D). This indicates that (i) translation of TimP starts at the mutated ATG codon and (ii) the TimP protein, but not the *timP* mRNA, is toxic upon overexpression.

10.1128/mBio.01659-20.7 FIG S3 ORF discovery from *ryfA/timP* mRNA alignment. Download FIG S3, EPS file, 0.2 MB. Copyright © 2020 Andresen et al. 2020 Andresen et al. This content is distributed under the terms of the Creative Commons Attribution 4.0 International license.

### TimP is an inner membrane protein.

**FIG 3:**
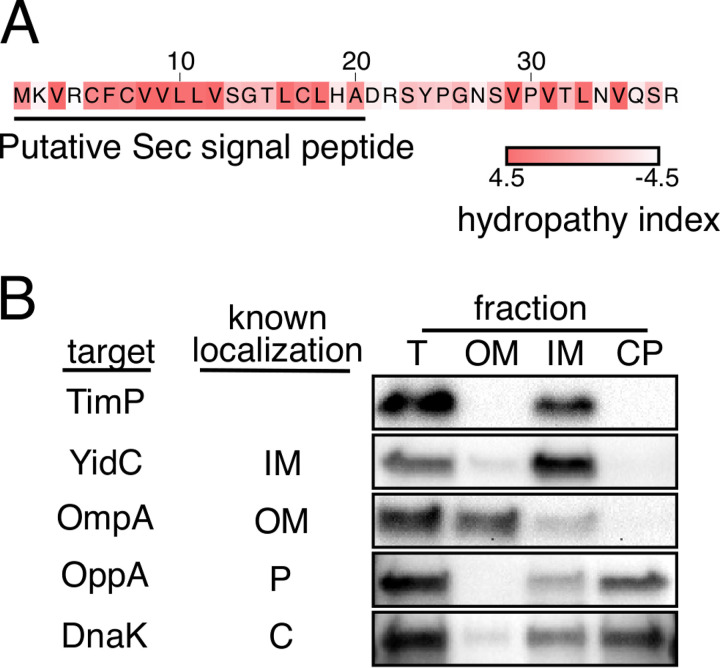
The small protein TimP carries a putative Sec system signal sequence and is targeted to the inner membrane. (A) TimP sequence in *Salmonella* serovar Typhimurium strain SL1344. The hydrophobicity of each amino acid residue is colored according to the Kyte-Doolittle hydropathicity scale (79). (B) Western blot analysis after cell fractionation of a *Salmonella* strain expressing TimP-6×His. Antibodies against proteins with known cellular localization were used to verify fractionation efficiency. T, nonfractionated sample; OM, outer membrane; IM, inner membrane; CP, cytoplasm and periplasm.

TimP is a 38-amino-acid-long hydrophobic protein, with the majority of hydrophobic residues located within its N-terminal part (Fig. 3A). We analyzed the TimP sequence for a putative secretion system signal sequence using three different prediction tools: SignalP-5.0 (41), PRED-TAT (42), and Phobius (43). With high probability (*P* = 0.99 to 1.0), all three tools predicted a Sec translocase signal sequence spanning amino acids 1 to 20 (Table S3). SignalP-5.0 in addition predicted a signal peptidase I cleavage site between Ala20 and Asp21. However, Western blot analysis did not reveal cleavage products of TimP-6×His but only the full-size protein (Fig. 2B), indicating that the signal peptide is not cleaved off. The TimP signal sequence is predicted to be cleaved by signal peptidase I, whose activity requires a short-chain amino acid in positions −1 and −3 from the cleavage site to lock the substrate into its active site (44). While TimP carries a small amino acid (Ala) in position −1 from the predicted cleavage site, it has Leu in position −3, which is unfavorable for cleavage. Without signal sequence removal, proteins can be transported across, but not released from, the inner membrane (45). Indeed, when we fractionated cells expressing TimP-6×His, we detected the protein in the inner membrane fraction together with the control protein YidC (Fig. 3B). Thus, TimP is a toxic inner membrane protein carrying a signal sequence, suggesting Sec-dependent localization.

### TimP expression leads to membrane damage.

**FIG 4:**
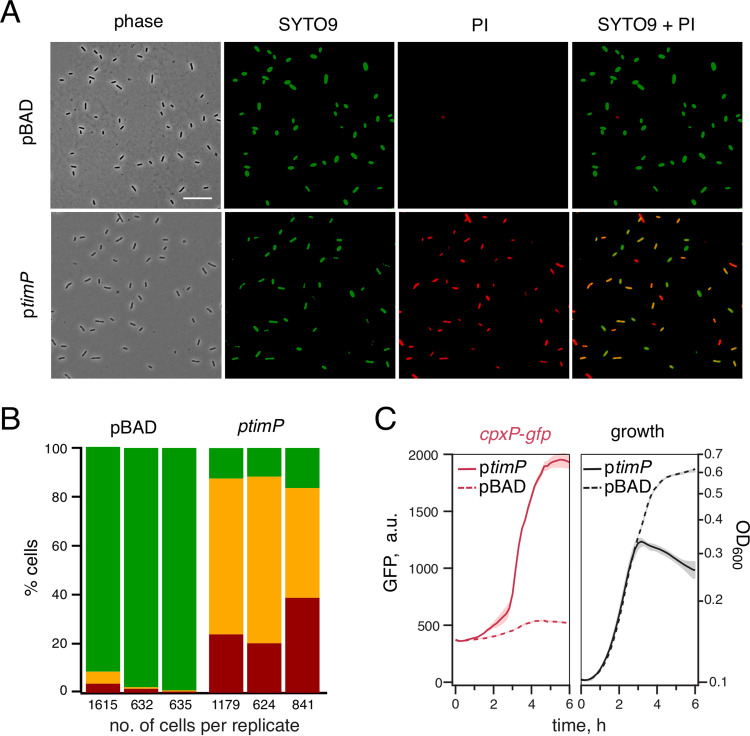
*timP* overexpression leads to leaky membranes. (A) *Salmonella* cells carrying either the vector control (pBAD33) or a *timP* overexpression construct (pYMB023) were grown in M9-based medium to exponential growth phase. At an OD_600_ of 0.35, *timP* expression was induced from a plasmid by addition of 0.2% l-arabinose. After 1 h of induction, cells were stained with SYTO9 and propidium iodine (PI), DNA dyes that do (SYTO9) or do not (PI) pass intact cell membranes. Phase contrast and fluorescence microscopy images of the stained cells are provided. Scale bar, 15 μm. (B) Quantification of stained cells (as in panel A) from three independent experiments. Coloring indicates cells that contain SYTO9 (green), propidium iodine (red), or both of the dyes (yellow). Numbers below stacked bars indicate the number of cells analyzed for each replicate. (C) Cells carrying a P*cpxP*-*gfp* reporter construct (pYMB011) were grown in the presence (p*timP*) or in the absence (pBAD) of an inducible *timP* expression construct (pYMB016). Cells were grown in LB containing 0.2% l-arabinose. Lines indicate average values from three independent transformants measured in two technical replicates. Shading below the lines indicates standard deviations across the measurements.

Small toxic proteins, such as TisB and Hok, are known or proposed to form pores in the inner membrane, causing membrane leakage (32, 46). One exception to this is the Bacillus subtilis inner membrane toxin BsrG, which rather than affecting membrane permeability induces aberrant membrane topology with continuous invaginations of the membrane (33). To investigate if TimP affects cell morphology and/or membrane permeability, we studied *timP*-expressing cells using microscopy. As judged by phase contrast imaging, 1 h of *timP* induction did not result in any observable morphological differences from a strain carrying a vector control (Fig. 4A), despite having a strong effect on growth (Fig. 1C). In contrast, when we analyzed the same samples for propidium iodine permeability, 86% (±2.5%) of the cells overexpressing *timP* were permeable to the dye, in comparison to 3.7% (±4.2%) for control cells (Fig. 4A and B). Hence, *timP* overexpression directly or indirectly confers a leaky-membrane phenotype. A study by Fozo and others showed that transient overexpression of the toxins IbsC, ShoB, LdrD, and TisB induces expression of the *cpxP* gene (47). CpxP is one of the most highly expressed members of the Cpx stress response, which is activated upon cell envelope stress (48). In accordance with TimP damaging the inner membrane, a transcriptional fusion between the *cpxP* promoter and the green fluorescent protein (GFP) gene was strongly activated upon *timP* overexpression (Fig. 4C). The induction of P*cpxP-gfp* preceded the decline in optical density, indicating that inner membrane damage occurs prior to growth inhibition. However, activation of the Cpx system is not required for TimP-dependent growth inhibition, as TimP toxicity is maintained in a strain lacking CpxR, the master transcriptional regulator of the Cpx system (Fig. S4).

10.1128/mBio.01659-20.8 FIG S4 Cpx system activation is not required for TimP toxicity. Wild-type and Δ*cpxR* strains were transformed with a *timP* overexpression construct (pYMB023) or the corresponding vector control (pBAD33). Cells were grown in the presence of 0.2% l-arabinose in M9-based minimal medium. Bacterial growth was measured in early stationary phase (8 h after inoculation). Bars indicate the average values from three independent transformants ± SD. Download FIG S4, EPS file, 0.1 MB. Copyright © 2020 Andresen et al. 2020 Andresen et al. This content is distributed under the terms of the Creative Commons Attribution 4.0 International license.

### Expression of TimP is inhibited by sRNA TimR.

**FIG 5:**
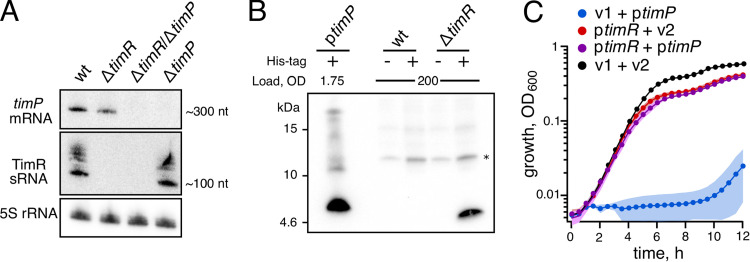
TimR inhibits *timP* expression and counteracts TimP-dependent toxicity. (A) Northern blot analysis of RNA extracted from the indicated *Salmonella* strains cells grown in LB to an OD_600_ of 0.4. The multiple bands detected when using a probe against TimR may indicate that this RNA is processed. The indicated approximate lengths of *timP* mRNA and TimR were determined using a logarithmic function based on the measured length from the wells of the gel to each band of the size marker. (B) Western blot analysis of *Salmonella* wild-type and Δ*timR* strains harboring a His tag in the native *timP* locus grown in LB medium to an OD of 2. Prior to gel loading, the TimP-6×His protein was concentrated using Ni-based purification. A sample from the TimP-6×His overexpression strain served as reference. The asterisk indicates an unspecific signal and serves as a loading control. (C) Growth of a *Salmonella* Δ*timP* Δ*timR* strain harboring plasmids overexpressing *timP* and/or *timR*. Data points show averages from 3 independent transformants. The shaded areas represent standard deviation. wt, wild type; v1 and v2, empty vectors.

Expression of small toxic proteins is generally heavily repressed, and activated only under specific stress conditions. For instance, transcription of the *tisB* gene is tightly repressed by transcription factor LexA, while translation of the *tisB* mRNA is inhibited both by an antisense RNA and by an intrinsic mRNA structure (49). In contrast to *tisB*, the *timP* mRNA is expressed at fairly high levels under all conditions tested in the SalCom gene expression compendium (50), suggesting that it is not strongly repressed at the transcriptional level. Indeed, natively expressed *timP* mRNA is readily detected by Northern blotting in cells growing exponentially in LB medium (Fig. 5A). Conversely, Western blot analysis of a wild-type strain in which the native *timP* ORF was tagged with a histidine tag failed to detect the protein (Fig. 5B). Similarly, a previous study failed to detect natively expressed sequential peptide affinity-tagged TimP (39). Apparently, although the *timP* mRNA is abundant, it is poorly translated, indicative of an inhibitory posttranscriptional mechanism. The *timP* gene is flanked by the uncharacterized sRNA gene *STnc2070*, here renamed *timR* (Fig. 1A). The TimR homolog in Shigella dysenteriae, RyfB1, was previously shown to decrease RNA levels of the *timP* homolog RyfA1 when overexpressed from a plasmid (37). The same study predicted a direct interaction between RyfA1 and RyfB1 RNAs, but no experimental evidence for the interaction was provided. In order to test whether TimR affects *timP* expression in *Salmonella*, we analyzed *timP* mRNA and TimR levels by Northern blotting and TimP levels by Western blotting. Northern analysis showed that deletion of either gene did not substantially affect the expression of the other (Fig. 5A). However, the *timR* deletion resulted in strongly increased levels of the TimP-6×His protein, indicating that (i) TimP is expressed not only when overexpressed but also from its native locus, and (ii) the TimR sRNA negatively affects the translation of TimP (Fig. 5B). Of note, the overexpression construct yielded ∼350-times-higher TimP levels than native expression upon *timR* deletion (Fig. 5B). In accordance with TimR repressing TimP production, a plasmid constitutively overexpressing TimR completely abrogated the toxicity of the TimP overexpression construct (Fig. 5C).

### TimR binds directly to the *timP* mRNA to inhibit translation.

**FIG 6:**
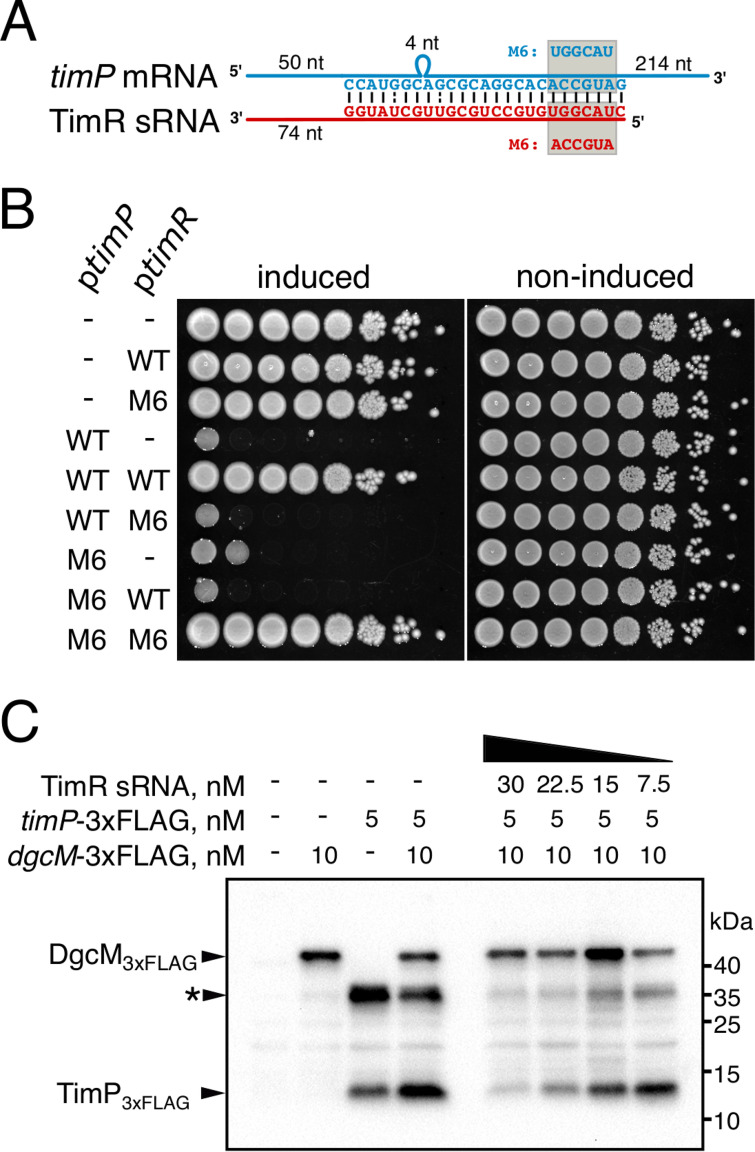
TimR inhibits *timP* translation by direct RNA-RNA interaction. (A) Predicted complementary sequence between *timP* mRNA and TimR sRNA. M6, mutations introduced into the *timP* and *timR* overexpression constructs assayed in panel B. (B) *timR* and *timP* genes with or without M6 mutations were cloned under P*lac* and P*araBAD* promoters in compatible plasmids. These genes were expressed either independently or combined in *Salmonella* cells by spotting dilutions of bacterial overnight cultures (grown without l-arabinose) onto LB plates with (induced) or without (noninduced) l-arabinose (0.2% final concentration). (C) TimP-3×FLAG (target) and DgcM-3×FLAG (control) proteins were synthesized in a cell-free translation system using the respective mRNAs as the templates. TimR was added to the samples prior to the translation mix, where indicated. Translation products were analyzed by Western blotting using an anti-FLAG antibody. The asterisk indicates a large protein product, which may represent TimP oligomers or TimP in complex with components of the *in vitro* translation kit.

To test if TimR can bind directly to the *timP* mRNA, we used the IntaRNA algorithm (51) to search for complementary sequences. This revealed an 18-nt-long continuous stretch of complementarity between the TimR 5′ region and the 5′UTR of the *timP* mRNA, indicating that these RNAs may interact *in vivo* (Fig. 6A; Fig. S5). To test this, we mutated the predicted interaction sites in the *timR* and *timP* overexpression constructs so that complementarity was restored when the two mutants were combined (Fig. 6A). Tenfold dilutions of overnight cultures expressing combinations of wild-type and mutant TimR/*timP* pairs were spotted on agar plates containing l-arabinose to induce *timP* expression (Fig. 6B). While wild-type TimR fully rescued cells from TimP toxicity, mutant/wild-type combinations were toxic (TimR-M6/*timP* and TimR/*timP*-M6). However, combining the two mutants, thereby restoring complementarity, also restored TimR-dependent rescue from toxicity. These results strongly indicate that TimR base pairs to the predicted region in *timP* mRNA *in vivo*, which leads to inhibition of TimP synthesis. To test this explicitly, we performed *in vitro* translation assays using a *timP*-*3×flag* mRNA in the presence or absence of TimR. Increasing concentrations of TimR specifically decreased the rate of TimP-3×FLAG synthesis, whereas translation of an unrelated mRNA (*dgcM*-*3×flag*) increased or was unaffected (Fig. 6C). We conclude that TimR is an antisense-type sRNA that binds to a complementary region in the 5′UTR of *timP* mRNA to inhibit translation.

10.1128/mBio.01659-20.9 FIG S5 (A) Predicted secondary structure of the *timP* mRNA generated by the Mfold algorithm. The TimR binding site, Shine-Dalgarno sequence, and TimP ORF are highlighted. (B) Sequence-structure alignment of the first 200 nucleotides of the *timP* mRNA generated by http://genome.ku.dk/resources/war/. Download FIG S5, PDF file, 1.6 MB. Copyright © 2020 Andresen et al. 2020 Andresen et al. This content is distributed under the terms of the Creative Commons Attribution 4.0 International license.

### Native expression of TimP induces low levels of membrane stress but does not affect growth.

**FIG 7:**
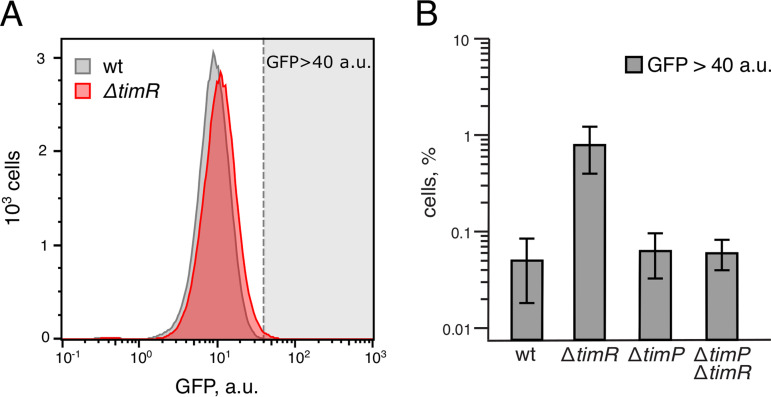
Induction of TimP expression upon *timR* deletion results in TimP-dependent *cpxP* promoter activation. (A) Flow cytometry analysis of *Salmonella* wild-type and Δ*timR* strains carrying a P*cpxP*-*gfp* transcriptional reporter during growth in LB medium to an OD_600_ of 2. (B) Fractions of cells in wild-type, Δ*timR*, Δ*timP*, or Δ*timP* Δ*timR* cultures that have higher expression of P*cpxP*-*gfp* than the set threshold (GFP > 40 arbitrary units [a.u.]). Bars indicate the average of results from two independent biological replicates ± SD.

The data presented in Fig. 1 indicate that overexpression of TimP is highly toxic and leads to irreversible growth inhibition. However, the condition(s) under which native TimP may be induced are unknown. Expression of TimR largely rivals that of *timP* mRNA under all conditions tested in the SalCom compendium (50), suggesting that under those conditions TimP synthesis should be repressed. The *timP* mRNA appears to be more stable than TimR, as judged by a rifampicin experiment (Fig. S6A), suggesting that a condition in which transcription of *timR* is repressed would allow translation of the more stable *timP* mRNA. Lacking a natural TimP-inducing condition, we used the Δ*timR* strain as a proxy for endogenous TimP induction. Interestingly, although a *timR* deletion allows *timP* to be translated (Fig. 5B), it does not affect cell growth as monitored by measuring optical density (Fig. S6B to D). While this indicates that low levels of TimP do not lead to severe toxicity, there may still be more subtle effects on cell physiology. To test this, we used the transcriptional P*cpxP-gfp* fusion, which is strongly activated upon TimP overexpression (Fig. 4C). Using single-cell measurements, we could detect a small, but significant, increase in *cpxP-gfp* expression in the Δ*timR* strain compared to that in a wild-type strain (Fig. 7A). The shift in GFP levels detected in the Δ*timR* strain was restored to wild-type levels upon additional deletion of *timP* (Fig. 7B), indicating that *cpxP* activation was dependent on *timP*.

10.1128/mBio.01659-20.10 FIG S6 (A) Wild-type *Salmonella* cells were grown in SPI-2 medium to an OD_600_ of 0.3. Transcription was stopped by addition of rifampicin to the growth medium. The decay of *timP* mRNA and TimR sRNA was estimated by Northern blotting of total RNA collected before and at the indicated times after rifampicin addition. (B to D) Growth curves of the indicated *Salmonella* strains grown in LB medium (B), M9 minimal medium supplemented with 0.4% glycerol and 0.1% Casamino Acids (C), or SPI-2 medium (D). Optical density at 600 nm was monitored during growth in 96-well plates. (E to G) Phenotypic analysis of *timP* and *timR* deletion strains. (E) The biofilm-associated phenotype rdar was investigated by spotting bacterial cultures on either regular LB plates or low-salt LB plates containing Congo red. The rdar phenotype was imaged after 3 days of incubation at 28°C. The *cspCE* deletion strain served as a positive control for impaired rdar morphology. (F) Persister cell formation was investigated by exposing bacteria growing exponentially in M9-glucose medium to a high dose (100 times the MIC) of ciprofloxacin. Samples withdrawn 3 and 5 h after ciprofloxacin addition were diluted and plated on antibiotic-free LB plates. The persister frequency was obtained by comparing CFU counts obtained before and after antibiotic treatment. Bars indicate averages from three biological replicates. Individual values are shown as dots. (G) The sensitivity of TimP-TimR system mutants to P22 bacteriophage was compared to that of the *ΔflhDC* strain. Overnight cultures were diluted in LB medium to an OD of 0.01 and grown aerobically for 1 h. P22 was added to the cultures, and numbers of PFU per milliliter were determined 5.5 h postinfection. Bars indicate averages from three biological replicates. Individual values are shown as dots. Download FIG S6, EPS file, 1.6 MB. Copyright © 2020 Andresen et al. 2020 Andresen et al. This content is distributed under the terms of the Creative Commons Attribution 4.0 International license.

### *timP*-TimR is also encoded by other enterobacteria.

**FIG 8:**
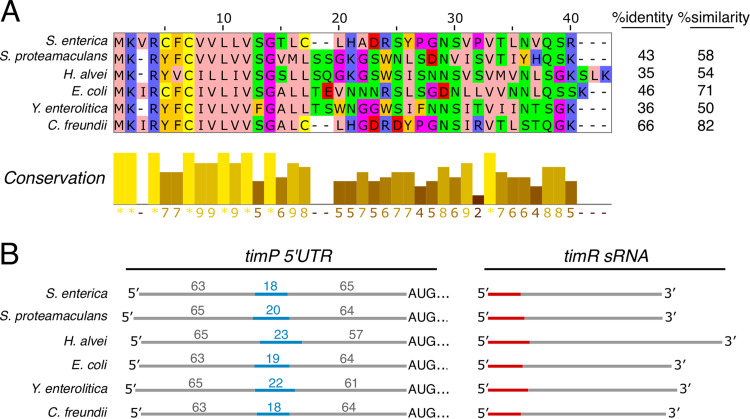
Conservation of TimP-TimR in enterobacteria. (A) TimP amino acid sequence conservation in different enterobacterial species. The multiple-sequence alignment was visualized using Jalview, and amino acid residues are colored according to the similarity of their physicochemical properties (Zappo coloring [80]). (B) *timP*-bearing enterobacterial species encode a TimR sRNA homolog which shares extensive complementarity between its 5´end (red) and a stretch of nucleotides in the 5′UTR of *timP* mRNA (blue).

According to Rfam, homologs of the *timP* RNA are present in many enterobacterial species (RF00126). The presence of in-frame start and stop codons indicates that all these sequences have the potential to encode homologs of TimP (Fig. S3). An alignment of TimP amino acid sequences revealed that the N-terminal part possessing the signal sequence is more conserved than the C-terminal region (Fig. 8A). To see if *timP* genes are generally flanked by an sRNA, as in the case of TimR in *Salmonella*, we searched homologs upstream of *timP* for complementary sequences. Strikingly, in all analyzed species, sequences complementary to the respective *timP* 5′ untranslated region (5′UTR) were found upstream of, and on the opposite strand from, each *timP* gene. The complementary sequences were followed by intrinsic terminators, suggesting that they, as TimR, represent antisense RNAs and inhibitors of the flanking *timP* gene. In addition, the location of the TimR interaction sites relative to those of *timP* ORFs is highly conserved between species (Fig. 8B). Thus, regulation of *timP* expression by TimR-like sRNAs appears to be a shared feature throughout enterobacteria.

## DISCUSSION

To date, at least 19 type I TA modules in E. coli have been described (28). In *Salmonella* strain SL1344, the subject of the current study, only seven type I TA systems are known (52). The higher number of TA loci in bacterial genomes often coincides with the magnitude of changes in the surrounding environment, depending on the lifestyle of the species (53–55). As *Salmonella* encounters dynamic environmental changes both inside and outside the host, the low number of type I TA systems identified in this organism is likely to be an underestimate. Identification of type I TA systems is hampered by the same problems as identification of small protein genes in general: toxin genes are not annotated in genomes because of their small size and the low sequence conservation of the protein product. Compared to other small proteins, type I toxins are even more challenging to identify experimentally. Toxin expression is strongly repressed under common laboratory conditions, resulting in (i) toxin deletion strains lacking obvious phenotypes and (ii) escape from approaches that rely on protein expression, such as proteomics, immunoblotting, and ribosome profiling.

In this study, we describe the *Salmonella* serovar Typhimurium *timR-timP* locus, which is reminiscent of type I TA modules in terms of the following. (i) Its gene arrangement is similar to those of *shoB-ohsC*, *tisB-istR1*, *zor-orz*, and *dinQ-agrB* in E. coli (29), as *timR* and *timP* are divergently transcribed from directly adjacent genes. (ii) Overexpression of *timP* is toxic to the bacterial cell, a general feature of all TA system toxins (28). (iii) *timP* mRNA translation is repressed by an antisense sRNA, which is applicable to all type I TA systems (30). (iv) TimR is less stable than *timP* mRNA (Fig. S6A), potentially allowing TimP expression upon stresses which lead to repressed *timR* transcription. (v) TimP overexpression entails membrane damage, the most common outcome of type I toxin overexpression (28). However, despite these obvious similarities to type I TA systems, the TimR/P system displays some important differences. First, mRNA processing is required for efficient translation of many type I toxin mRNAs, including *tisB*, *hok*, *zorO*, *shoB*, *dinQ*, and *aapA1* (30, 56–61). In contrast, the facts that the full-length mRNA, but no shorter isoforms, is detected by Northern blotting analysis (Fig. 5A) and that full-length *timP* mRNA is efficiently translated *in vitro* (Fig. 6C) suggest that mRNA processing is not required for TimP expression. Second, although TimP localizes to the cytoplasmic membrane, as do the majority of the type I toxins, it may depend on a different mechanism. TimP carries a predicted Sec system signal sequence at its N terminus, suggesting that it uses the Sec translocon for membrane insertion. All known membrane-targeted type I toxins lack a signal sequence and are inserted through their characteristic transmembrane domains. This may indicate that TimP has a different mechanism of action from, e.g., those of HokB and TisB toxins, which form pores in the lipid bilayer (32, 46).

One important question is what biological function(s) TimP possesses. There is a wide range of functions described for membrane-bound small proteins in bacteria, whereas the biological functions for type I TA systems are less clear (31). As mentioned above, functions of type I toxins are challenging to study, since toxin expression is repressed under normal growth conditions. For this reason, many studies on TA systems have been conducted using ectopic toxin expression, often resulting in cellular toxin concentrations that by far exceed what likely could ever be reached through endogenous expression. In line with this, it has been proposed that toxins can inhibit growth or kill cells in a dose-dependent manner (62). This then raises concerns about whether the small proteins encoded by TA systems act as toxins when expressed from their native loci. Notably, toxicity due to overexpression is not uncommon for proteins with well-characterized cellular functions not related to toxicity. With that said, some type I TA systems have been shown to contribute to important physiological processes, including persister cell formation, survival upon UV damage, and recycling of damaged RNA produced under SOS stress conditions (34, 59, 62, 63). Overexpression of *timP* from an inducible promoter causes growth inhibition and membrane leakage. This may be due to an evolved function or due to nonphysiological effects achieved by overexpression (e.g., by disrupting the inner membrane due to overcrowding with a hydrophobic protein, by TimP aggregation [Fig. 2B and 5B], by jamming of the Sec translocon, or through adverse effects on putative interaction partners), which consequently leads to a systemic response. Therefore, at this point, we refrain from speculating on TimP’s biological function based on our overexpression experiments, mainly because our data indicate that relieving endogenously expressed *timP* mRNA from TimR repression permits TimP translation, however without an apparent effect on growth (Fig. S6B-D). What putative roles could TimP have for bacterial physiology and/or survival? A *timP* homolog in an ocular pathogen (E. coli strain L-1216/2010) was previously shown to affect biofilm formation by affecting production of curli fimbriae and cellulose nanofibers (38). However, deleting one or both components of the *timPR* system in *Salmonella* did not affect the biofilm-dependent rdar (red, dry, and rough) morphotype (Fig. S6E), indicating that biofilm formation is not a universal phenotype related to *timPR* systems in different bacteria. Regarding other phenotypes previously associated with type I TA systems, we could not observe a significant effect of *timR* and/or *timP* deletions on either persister cell formation or P22 bacteriophage infection (Fig. S6F and G). Although we did not find a clear phenotype for *tim* mutants in *Salmonella*, our Cpx envelope stress reporter (P*cpxP-gfp* fusion) results indicated a mild stress in the *timR* deletion strain, suggesting that chromosomal expression of *timP* may have physiologically relevant effects on the bacterium. We anticipate that future studies will shed light on the physiological function(s) of TimP and clarify whether these rely on its toxic activity.

One route toward understanding the physiological context in which TimP may play a role is to identify conditions which promote its expression. In *Salmonella*, the *timP* mRNA is upregulated in macrophages and host cell mimicking conditions (50), strongly repressed by (p)ppGpp and one of the few detectable transcripts after long-term starvation and desiccation (64, 65). This hints at the *timPR* system being responsive to stress. However, since translation of TimP is controlled by TimR, transcriptomic data on *timP* mRNA levels alone may be a poor indicator of TimP expression. Screening approaches that monitor TimP/TimR expression under many different growth conditions, preferentially in single cells or that identify regulatory factors, may help us to understand when and how TimP is expressed to exert its function.

Another important issue concerns how TimR controls TimP expression. Overexpression of TimR abolishes TimP-dependent toxicity, and deletion of *timR* induces TimP expression (Fig. 5). The TimR 5′ end is complementary to the *timP* 5′UTR, and mutations within the complementary sequences of either RNA abolishes TimR-dependent rescue from TimP toxicity (Fig. 6). In a cell-free translation system, TimR inhibits translation of *timP* mRNA but not of an unrelated control mRNA (Fig. 6). Taken together, these results strongly suggest that TimR is an antisense-type sRNA that inhibits translation by binding to the *timP* 5′UTR. How does this work mechanistically? The TimR binding site is located far upstream (>60 nucleotides) of the *timP* ribosome-binding site (RBS) (Fig. 2A and 8; see also Fig. S5), ruling out direct occlusion of 30S binding at the RBS as a possible mechanism of regulation. Several cases where base pairing sRNAs inhibit translation by binding far upstream of an RBS have been described. A classic example is the inhibition of *repA* translation by CopA RNA in copy number control of plasmid R1 (66). CopA targets the RBS of a small upstream ORF to inhibit translation initiation, which is required for initiation at the *repA* RBS through translational coupling (67). The *timP* 5′UTR harbors a short ORF preceded by a Shine-Dalgarno-like sequence, suggesting that a CopA-like mechanism might be applicable. However, the lack of conservation of the upstream ORF challenges this hypothesis. Another example is the *tisB* mRNA, which harbors a highly structured RBS that is inaccessible for direct 30S entry (68). Here, a single-stranded region far upstream acts as a ribosome standby site that allows transient 30S binding followed by relocation to the RBS (56, 69). The cognate antisense sRNA IstR-1 targets the standby site, thereby inhibiting translation initiation (56, 70). A similar mechanism ensures translation initiation at the structured RBS of the *zorO* mRNA (58). A recent study showed that the *manY* mRNA contains an upstream translational enhancer, at which ribosomal protein S1 associates to promote translation initiation at the RBS (71). The sRNA SgrS inhibits translation by targeting the enhancer sequence. Our current data are compatible with any mechanism in which translation initiation at the *timP* mRNA requires an upstream element overlapping the TimR binding site. However, other mechanisms, for instance involving a TimR-dependent structural alteration of the *timP* mRNA, should also be considered.

In summary, we have identified a genetic module in *Salmonella* which shares similarities with type I TA systems. Further research will show if this system is important for regulation of the bacterial growth rate or has a toxicity-independent function in the bacterial membrane.

## MATERIALS AND METHODS

### Bacterial strains and growth conditions.

Salmonella enterica subsp. *enterica* serovar Typhimurium strain SL1344 was used throughout the study as the wild-type strain and as a parent strain for construction of chromosomal *tim* deletions (72). Bacteria were grown aerobically at 37°C in LB medium or in M9 minimal medium supplemented with 0.1% Casamino Acids and either 0.2% glucose or 0.4% glycerol as the carbon source (73). Where indicated, 0.2% l-arabinose was added to induce *timP* expression. For plasmid maintenance, ampicillin (100 μg/ml) or chloramphenicol (15 μg/ml) was added to the growth medium. For growth curve experiments, cells were grown in 96-well plates. Cultures were shaken, and optical density at 600 nm (OD_600_) was measured at 5-min intervals. OD_600_ values were normalized to that of the growth medium control. For spotting assays, a 10-fold dilution series of cell suspensions was prepared in phosphate-buffered saline (PBS). Four microliters of each dilution was spotted on LB plates or LB plates containing 0.2% l-arabinose and incubated overnight at 37°C prior to being imaged.

### Molecular cloning and strain construction.

Plasmids and oligonucleotides used in this study are listed in Tables S1 and S2 in the supplemental material, respectively. The construction of *timP* and *timR* overexpression vectors is described in Table S1. Tim system deletion mutants were constructed using Lambda red-mediated homologous recombination (74) and oligonucleotides EHO-1346 and EHO-1349 for *timR* deletion, EHO-1347 and EHO-1348 for *timP* deletion, and EHO-1346 and EHO-1347 for *timP/timR* double deletion (Table S2). TimP was hexahistidine tagged in the chromosome using scarless mutagenesis (75) with EHO-1516. All chromosomal mutations were transferred into a clean background using P22 bacteriophage-mediated transduction, as described in Text S1.

10.1128/mBio.01659-20.1 TEXT S1 Supplementary materials and methods. Download Text S1, DOCX file, 0.03 MB. Copyright © 2020 Andresen et al. 2020 Andresen et al. This content is distributed under the terms of the Creative Commons Attribution 4.0 International license.

10.1128/mBio.01659-20.2 TABLE S1 Plasmids and plasmid construction. RE cloning, plasmid construction using restriction enzymes; OE-PCR, overhang extension-PCR with overlapping primers. Download Table S1, PDF file, 0.04 MB. Copyright © 2020 Andresen et al. 2020 Andresen et al. This content is distributed under the terms of the Creative Commons Attribution 4.0 International license.

10.1128/mBio.01659-20.3 TABLE S2 Oligonucleotides used in this study. Download Table S2, PDF file, 0.04 MB. Copyright © 2020 Andresen et al. 2020 Andresen et al. This content is distributed under the terms of the Creative Commons Attribution 4.0 International license.

10.1128/mBio.01659-20.4 TABLE S3 TimP signal peptide predictions. SPase, signal peptidase. Download Table S3, PDF file, 0.03 MB. Copyright © 2020 Andresen et al. 2020 Andresen et al. This content is distributed under the terms of the Creative Commons Attribution 4.0 International license.

### Subcellular fractionation.

*ΔtimP* cells carrying the TimP-6×His expression vector (pLA208) were grown in M9-glycerol medium until an OD_600_ of 0.3 was reached. Expression was induced with 0.2% l-arabinose. After 15 min of induction at 37°C, cells from two 50-ml cultures were harvested by centrifugation at 5,000 × *g* for 10 min at 4°C. Cells were washed once with PBS and stored at −80°C. The two pellets were thawed on ice, followed by resuspension in 1.5 ml of ice-cold PBS-E (1× PBS containing 5 mM EDTA). Cells were disrupted by sonication (3 times for 15 s each time). The sample volume was brought up to 5 ml with PBS-E. One of the samples was acetone precipitated to serve as a nonfractionated control, and the other sample was further fractionated as follows. Cell lysate was centrifuged at 100,000 × *g* for 1 h at 4°C. The collected supernatant contains cytoplasmic and periplasmic content. The pellet was resuspended in PBS-E and 2% sodium-lauryl sarcosinate, a detergent that specifically dissolves the inner membrane (76). After 30 min of incubation at room temperature, the suspension was centrifuged at 100,000 × *g* for 15 min at 4°C to separate the inner membrane fraction (supernatant) from the outer membrane fraction (pellet). Pellets were resuspended in 1 ml lysis buffer containing 5 mM EDTA (see “Enrichment of TimP-6×His” below). The inner membrane and soluble fraction were acetone precipitated and dissolved in lysis buffer with 5 mM EDTA.

### Microscopy.

Strains were grown to an OD_600_ of 0.3 in M9-glycerol medium. Expression of *timP* was induced with 0.2% l-arabinose for 1 h, and cells were stained with SYTO and propidium iodide (PI) dyes using the LIVE/DEAD kit according to the manual (Invitrogen). Stained cells were trapped in agarose pads and imaged using a fluorescence microscope (×100 magnification; Nikon Eclipse Ti). Quantification of cells with permeable and nonpermeable membranes was done using ImageJ software.

### Northern blotting.

RNA extraction and Northern blotting were performed as described in reference 77, with the exception that Church buffer (78) was used to block nonspecific binding sites on the membrane. Sequences for the oligonucleotides used for *timP* mRNA (EHO-1344), TimR sRNA (EHO-1345), and control 5S rRNA (EHO-861) detection are in Table S2.

### Enrichment of TimP-6×His.

When indicated, TimP-6×His was enriched from protein samples prior to Western blotting as follows. Overnight cultures of strains carrying either the TimP-6×His overexpression plasmid (pLA208) or a *timP-6×His* allele in the native *timP* locus on the chromosome were diluted to an OD_600_ of 0.01 in fresh medium (M9 medium for pLA208, LB medium for chromosomal expression) and grown at 37°C with agitation at 220 rpm. For plasmid expression, when the cultures reached an OD_600_ of 0.4, l-arabinose was added to a final concentration of 0.2% for 15 min. For chromosomal expression, cells were harvested when the cultures reached an OD_600_ of 2.0. Bacterial cells were pelleted by centrifugation at 4,000 × *g* for 20 min at 4°C. Cell pellets or acetone-precipitated protein extracts were dissolved in Ni-nitrilotriacetic acid (NTA) lysis buffer (100 mM NaH_2_PO_4_, 10 mM Tris-HCl, 8 M urea, 0.05% Tween 20, pH 8) and incubated on an end-over-end roller for 1 h at room temperature. Twenty microliters of Ni-NTA magnetic agarose beads (Qiagen) were equilibrated in Ni-NTA lysis buffer, added to the sample (corresponding to 50 OD units in case of TimP-6×His expression from pLA208 and 800 OD units in case of chromosomal expression in the Δ*timR*/*timP*-6×His strain), and incubated end-over-end for 1 h at room temperature. Beads were washed four times with an equal volume of Ni-NTA wash buffer (100 mM NaH_2_PO_4_, 10 mM Tris-HCl, 8 M urea, 0.05% Tween 20, pH 6.3). Washed beads were boiled in 40 μl of Tricine-SDS-PAGE sample buffer for 5 min, and the supernatant was used for analysis by Western blotting.

### Western blotting.

Protein samples from subcellular fractionation experiments or Ni-NTA-based concentration experiments were resuspended in Tricine-SDS-PAGE loading buffer (3% SDS, 1.5% β-mercaptoethanol, 7.5% glycerol, 37.5 mM Tris-HCl, pH 7, 0.01% Coomassie blue G-250) and separated on Tricine-SDS-PAGE gels (Bio-Rad). Proteins were transferred on a 0.2-μm-pore-size polyvinylidene difluoride (PVDF) membranes using the TransBlot TURBO transfer system and preassembled transfer sandwiches (Bio-Rad). TimP-6×His was detected with HisProbe-HRP conjugate (ThermoFisher Scientific) and Amersham ECL Prime reagents according to the supplier’s protocols (GE Healthcare). FLAG-tagged proteins were detected using monoclonal anti-FLAG M2–peroxidase (Sigma-Aldrich).

### *In vitro* translation assay.

RNAs were *in vitro* transcribed (MEGAscript kit; Life Technologies) from a PCR-generated DNA template (*timP*-*3×flag*, oligonucleotides EHO-1421 and EHO-1422, PCR template pYMB025) or a template generated by Klenow fragment-dependent oligonucleotide fill-in (TimR RNA, oligonucleotides EHO-1419 and EHO-1420). *dgcM*-*3×flag* mRNA was produced as described previously (78). RNAs were purified by denaturing PAGE, followed by phenol extraction and ethanol precipitation. *In vitro* translation was performed with the PURExpress *in vitro* protein synthesis kit (New England BioLabs) as follows. *In vitro*-transcribed RNAs were denatured at 95°C for 5 min and cooled on ice. After addition of TMN buffer (final concentrations, 20 mM Tris, 5 mM Mg-acetate, 100 mM NaCl, pH 7.5) RNAs were renatured at 37°C for 5 min and mixed. For each *in vitro* translation reaction, 2 μl of component A, 1.5 μl of component B, and 1.5 μl RNA mix was incubated at 37°C for 20 min. Reactions were stopped with equal volumes of 2× Tricine-SDS-PAGE sample buffer on ice.

### GFP measurements.

*cpxP* promoter activity from reporter fusion construct P*cpxP-gfp* (pYMB011) was measured at the population level in cultures grown in LB medium in 96-well plates (excitation, 480 nm; emission, 520 nm). Single-cell GFP fluorescence was measured from cultures grown to an OD_600_ of 2 in LB medium using a MACSQuant VYB flow cytometer (channel B1, 488 nm/525/50 nm).
