# Supplementary figures and images for: The Small Toxic Salmonella Protein TimP Targets the Cytoplasmic Membrane and Is Repressed by the Small RNA TimR
Source: mBio. 2020 Nov 10;11(6):e01659-20. doi: 10.1128/mBio.01659-20 (PMC7667032; doi:10.1128/mBio.01659-20)

A

*timP* mRNA

- TimR binding site
- Shine-Dalgarno sequence
- Open reading frame

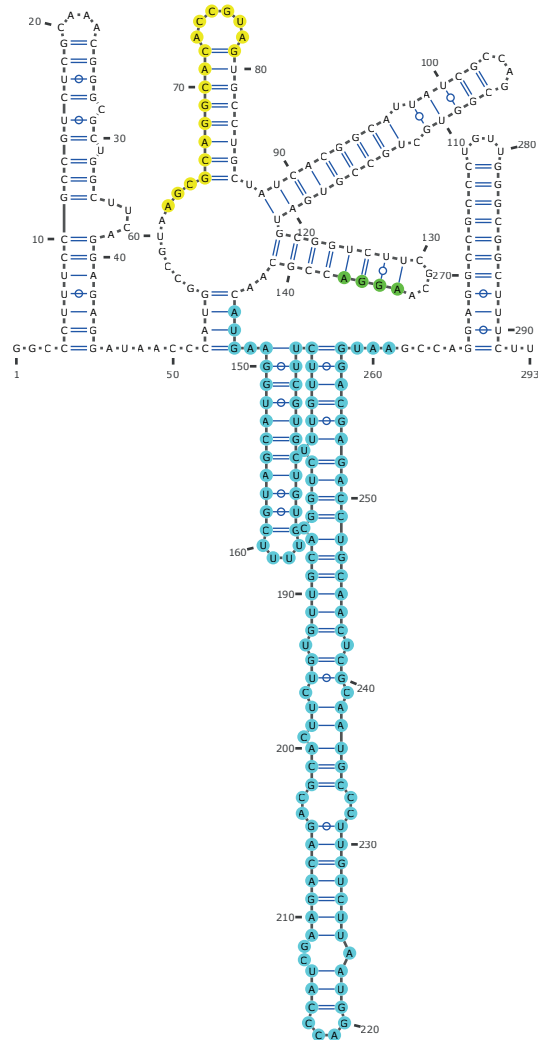

B

TimR binding site

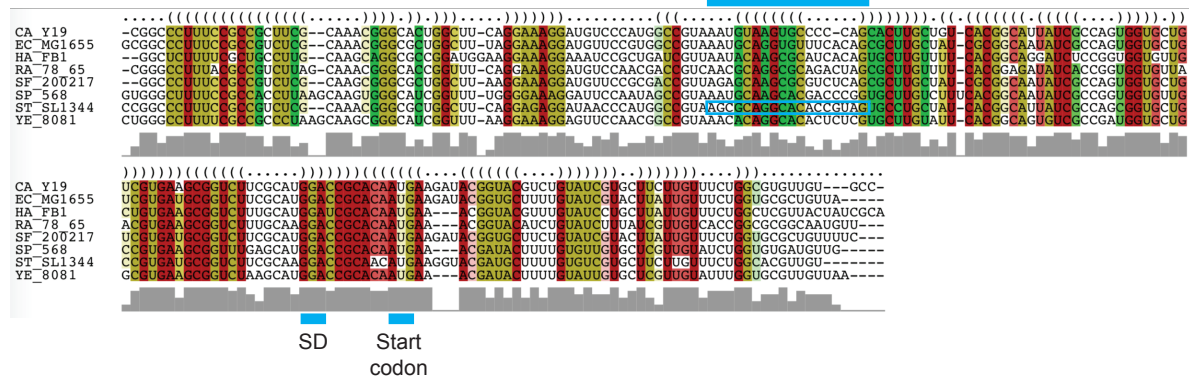

Supplement: FIG S5 [file mBio.01659-20-sf005.pdf]
