# Supplementary material for: The Small Toxic Salmonella Protein TimP Targets the Cytoplasmic Membrane and Is Repressed by the Small RNA TimR
Source: mBio. 2020 Nov 10;11(6):e01659-20. doi: 10.1128/mBio.01659-20 (PMC7667032; doi:10.1128/mBio.01659-20)
Supplement: TEXT S1 [file mBio.01659-20-s0001.docx]

**SUPPLEMENTARY MATERIALS AND METHODS**

*Growth in SPI-2 medium*. Where indicated, bacteria were grown in SPI-2 medium in the presence of low [Mg^2+^] (10 µl MgCl_2_) to mimic the host-cell environment (Kröger et al. 2013).

*Rifampicin experiment.* To estimate *timP* mRNA and TimR sRNA stability cells were grown in SPI-2 medium to OD_600_=0.3. The transcription was stopped by addition of 500 µg/ml rifampicin to the growth medium, and samples for Northern blotting were taken at indicated time intervals. The total RNA was extracted and analysed as described in *Northern blotting*.

*Persister assay.* Bacterial strains were streaked on agar plates and incubated overnight at 37°C. Single colonies were inoculated in 2 ml LB medium in 15 ml Falcon tubes and cultured for approximately 16 h at 37°C with 220 rpm shaking. Overnight cultures were diluted to OD_600_ 0.02 in 20 ml LB medium in 200 ml Erlenmeyer flasks. Diluted cells were grown in a water bath shaker (200 rpm) at 37°C until they reached exponential phase (OD_600_ 0.3; reached in our setup after ca 2 h). At this point, 2 ml of sample aliquots were taken for untreated cultures. Bacterial cultures were pelleted by centrifugation at 16,000 g for 5 min. The bacterial pellets were resuspended in 2 ml of sterile filtered PBS, serially diluted in PBS and plated (100 µl) on agar plates. In parallel, exponentially grown cultures were treated with a lethal concentration of ciprofloxacin (1 µg/ml) (100x MIC) for 3 h and 5 h. After antibiotic treatment, 2 ml of sample aliquots were taken and pelleted as described above. Bacterial pellets were resuspended in 200 µl sterile filtered PBS, serially diluted and plated to determine the number of antibiotic-surviving cells. All plates were incubated at 37°C for approximately 20 h and cfu/ml was determined from plates containing 30 – 300 bacterial colonies. The fraction of persister cells was calculated as the ratio of cfu/ml before and after antibiotic treatment.

*P22-mediated transduction.* Bacteriophage P22 was used to transduce chromosomal mutations into clean background, and to estimate the phage resistance of the *tim* system deletion strains. Phage lysates were prepared from the donor strains as follows. The overnight culture was diluted to OD 0.01 in 10 ml LB medium. After one hour of growth at 37ºC, culture was infected 1 µl of P22 stock solution (6x10^7^ pfu/ml) and grown for another 5.5 hours. Cells were lysed by vortexing the suspension in the presence of 12% (v/v) chloroform. Cell debris was removed with centrifugation at 16,000 g for 5 min. Phage titre of each lysate was determined by spotting ten-fold dilution series of the lysate on soft agar (0.3% agar in LB) containing ~10^7^ wild-type cells/ml. Plaque forming units per millilitre of lysate was calculated (pfu/ml) after overnight incubation of the plates at 37ºC. For transduction, 100 µl of recipient´s overnight culture was infected with 5 µl-s of donor lysate and incubated at room temperature for 20 minutes. Cells were centrifuged (16,000 g, 5 min), resuspended in 0.9% NaCl and plated on selective plates. Transductants were re-streaked on EBU plates (Bochner 1984) to allow for selection of P22-free strains and the insertion of the antibiotic resistance marker was confirmed by PCR.
