# Supplementary material for: The Small Toxic Salmonella Protein TimP Targets the Cytoplasmic Membrane and Is Repressed by the Small RNA TimR
Source: mBio. 2020 Nov 10;11(6):e01659-20. doi: 10.1128/mBio.01659-20 (PMC7667032; doi:10.1128/mBio.01659-20)
Supplement: TABLE S1 [file mBio.01659-20-st001.pdf]

name	description/source	construction						
		method	fragment 1			fragment 2		
			template	*oligo1*	*oligo2*	*template*	*oligo1*	*oligo2*
pBAD-TOPO	*Invitrogen*							
pBAD33	*Guzman et al., 1995*							
pUA66	*Zaslaver et al., 2006*							
pJV300	*Sittka et al., 2007*							
pYMB011	pUA66-PcpxP-gfp	PCR-> RE cloning (XhoI/BamHI)	SL1344 gDNA	EHO-1295	EHO-1296	pUA66 (XhoI/BamHI)		
pYMB016	pBAD-TOPO-timP	PCR-> RE cloning (blunt/EcoRI)	SL1344 gDNA	EHO-1224	EHO-1225			
pYMB016_mut	pYMB016 ATG->AAG	OE-PCR	pYMB016	EHO-1331	EHO-1332			
pYMB023	pBAD33-timP	PCR-> RE cloning (XbaI)	pBAD33	EHO-1356	EHO-1023	SL1344 gDNA	EHO-1355	EHO-1224
pYMB024	pBAD33-timP (ATG->AAG)	PCR-> RE cloning (XbaI)	pBAD33	EHO-1356	EHO-1023	pYMB016_mut	EHO-1355	EHO-1224
pYMB025	pBAD33-timP-3xFLAG	OE-PCR	pYMB023	EHO-1333	EHO-1334			
pLA201	pJV300-timR	PCR-> RE cloning (blunt/XbaI)	SL1344 gDNA	EHO-1357	EHO-1383			
pLA206	pBAD33-timP-1xFLAG	OE-PCR	pYMB023	EHO-1449	EHO-1450			
pLA207	pBAD33-timP-HA	OE-PCR	pYMB023	EHO-1451	EHO-1452			
pLA208	pBAD33-timP-6His	OE-PCR	pYMB023	EHO-1453	EHO-1454			
pEH792	pJV300-timR(M6)	PCR->ligation	pLA201	EHO-1414	PLlacO-C			
pEH795	pBAD33-timP(M6)	PCR-> ligation	pYMB023	EHO-1415	EHO-1416			
pLA218	pBAD33-timP(ATG->AAG)-6His	OE-PCR	pLA208	EHO-1331	EHO-1332			
