# Supplementary material for: The Small Toxic Salmonella Protein TimP Targets the Cytoplasmic Membrane and Is Repressed by the Small RNA TimR
Source: mBio. 2020 Nov 10;11(6):e01659-20. doi: 10.1128/mBio.01659-20 (PMC7667032; doi:10.1128/mBio.01659-20)
Supplement: TABLE S2 [file mBio.01659-20-st002.pdf]

Plasmid construction

oligo ID	sequence (5´-3´)
EHO-1023	TGGAGAAACAGTAGAGAGTTGCGA
EHO-1224	[Phos]GGCCCTTTCCGCCGTCTC
EHO-1225	GTTTTGAATTCTCTACGAGCGCCGCTGGG
EHO-1295	GTTTTCTCGAGGCTCTCGGTCATCATCAACT
EHO-1296	GTTTTGGATCCCTGTCGCGATTCAACGAGA
EHO-1331	TTGCGGTCCTTGCGAAGACCG
EHO-1332	GGACCGCAACAAGAAGGTACGATG
EHO-1333	GATCTTTATAATCGCCATCATGATCTTTATAATCCGCGCCCCTGCTCTGGACGTTGAGCGTTAC
EHO-1334	ATTATAAAGATCATGATATTGATTATAAAGATGATGATGATAAATGATAAGCCAGGAGGCCGCCCTGTTG
EHO-1355	GTTTTTCTAGAGAATTCTCTACGAGCGCCGCTGGG
EHO-1356	ATATATCTAGAAGATTAAATCAGAACGCAGA
EHO-1357	[Phos]CTACGGTGTGCCTGCGTTGC
EHO-1383	ATATATCTAGAGCGAATCTACATAAATTCTGTTTAAGCAGGC
EHO-1396	CACCTAGCCACACCCTGCGTTGCTATGGCAAC
EHO-1397	CGCAGGGTGTGGCTAGGTGCTCAGTATCTTGTTATCC
EHO-1398	CGCAGGGTGTGGCTAGTGCCTGCTATCACGGC
EHO-1399	GCACTAGCCACACCCTGCGCTTACGGCCA
EHO-1414	[Phos]CATGCCAGTGCCTGCGTTGCTATG
EHO-1415	[Phos]TGGCATGTGCCTGCTATCACGGC
EHO-1416	[Phos]GTGCCTGCGCTTACGG
EHO-1449	TTTATCATCGTCATCTTTATAATCCCTGCTCTGGACGTTGAGC
EHO-1450	GATTATAAAGATGACGATGATAAATGATAAGCCAGGAGGCCGC
EHO-1451	CATAATCCGGCACATCATACGGATACCTGCTCTGGACGTTGAGC
EHO-1452	GTATGATGTGCCGGATTATGCGTGCTGATAAGCCAGGAGGCCGC
EHO-1453	GTGATGATGGTGGTGATGCCTGCTCTGGACGTTGAGC
EHO-1454	CATCACCACCATCATCACTGATAAGCCAGGAGGCCGC
PLlacO-C	GTGCTCAGTATGTTGTTATCCG

Strain construction

oligo ID	sequence
EHO-1346	CTGATTTAAAGCGAATCTACATAAATTCTGTTTAAGCAGGGTGTAGGCTGGAGCTGCTTC
EHO-1347	CTGCGTGAAGACACAACGTGCCAGAAACAAGAAGCACGACcatatgaatatcctcctta
EHO-1348	TACCGGGAAACTATTCAATATTTGAGGTATTATCTTTCCCGTGTAGGCTGGAGCTGCTTC
EHO-1349	ATCATATTGACATTTGCTCCCTTAAAAGGGAGTATTAATCcatatgaatatcctcctta
EHO-1516	CTGAAGAAAAGAAAGCCGCCCAACAGGGCGGCCTCCTGGCTCATTAGTGGTGATGATGGTGGTGCCTGCTC TGGACGTTGAGCGTTACGGGAACAGAATTACCT

Northern blotting

oligo ID	sequence
EHO-1344	ACACAACGTGCCAGAAACAAGAAGCACGACACAAAAGCAT
EHO-1345	GGCCTTTCCAGCAGGCTTTGTTGCCATAGCAACGCAGGCACACCG
EHO-861	CTACGGCGTTTCACTTCTGAGTTC

***In vitro* translation templates**

oligo ID	sequence
EHO-1419	gaaattaatacgactcactataggCTACGGTGTGCCTGCGTTGCTATGGCAACAAAGCCTGCTGGAAAGGC
EHO-1420	CACAAAAAAACCGCTCAATTGAGCGGTTTTTTTGTGCTGGTCCGGTTCGCGGCCTTTCCAGCAGGCTTTGT
EHO-1421	gaaattaatacgactcactataGGCCCTTTCCGCCGTC
EHO-1422	AAGAAAAGAAAGCCGCCCAACA
