# Supplementary material for: The Small Toxic Salmonella Protein TimP Targets the Cytoplasmic Membrane and Is Repressed by the Small RNA TimR
Source: mBio. 2020 Nov 10;11(6):e01659-20. doi: 10.1128/mBio.01659-20 (PMC7667032; doi:10.1128/mBio.01659-20)
Supplement: TABLE S3 [file mBio.01659-20-st003.pdf]

	Signal peptide (SP)			Cleavage site (CS)	
	**Prediction**		**Probability**	**CS position**	**Probability**
**SignalP-5.0**	Sec SP (1-20 aa)		0.989995	[LHA-DR]	0.7891
		SPase I	0.865373		
		SPase II	0.124622		
**PRED-TAT (new model)**	Sec SP (1-20 aa)		1	[LHA-DR]	n/a
**PRED-TAT (old model)**	Sec SP (1-20 aa)		0.998	[LHA-DR]	n/a
**Phobius**	Sec SP (1-20 aa)		n/a	n/a	n/a
